# Improving microalgal growth by strengthening the flashing light effect simulated with computational fluid dynamics in a panel bioreactor with horizontal baffles†

**DOI:** 10.1039/c8ra02863j

**Published:** 2018-05-23

**Authors:** Qing Ye, Jun Cheng, Zongbo Yang, Weijuan Yang, Junhu Zhou, Kefa Cen

**Affiliations:** State Key Laboratory of Clean Energy Utilization, Zhejiang University Hangzhou 310027 China juncheng@zju.edu.cn +86 571 87951616 +86 571 87952889

## Abstract

Biological CO_2_ elimination by photosynthetic microalgae is a sustainable way to mitigate CO_2_ from flue gas and other sources. Computational fluid dynamics was used to simulate algal cell movement with an enhanced flashing light effect in a novel panel bioreactor with horizontal baffles. Calculation results showed that the light/dark (L/D) cycle period decreased by 17.5% from 17.1 s to 14.1 s and that the horizontal fluid velocity increased by 95% while horizontal baffles were used under a 0.02 vvm air aeration rate and a microalgal concentration of 0.85 g L^−1^. The probability of the L/D cycle period within 5–10 s increased from 27.9% to 43.6%, indicating a 56% increase when horizontal baffles existed. It was proved by experiments that the mass-transfer coefficient increased by 31% and the mixing time decreased by 13% under a 0.06 vvm air aeration rate when horizontal baffles were used, and the algal biomass yield increased by ∼51% along with the decrease in the L/D cycle period when horizontal baffles were used.

## Abstract 2

Biological CO_2_ elimination by photosynthetic microalgae is a sustainable way to mitigate CO_2_ from flue gas and other sources.

## Graphical abstract

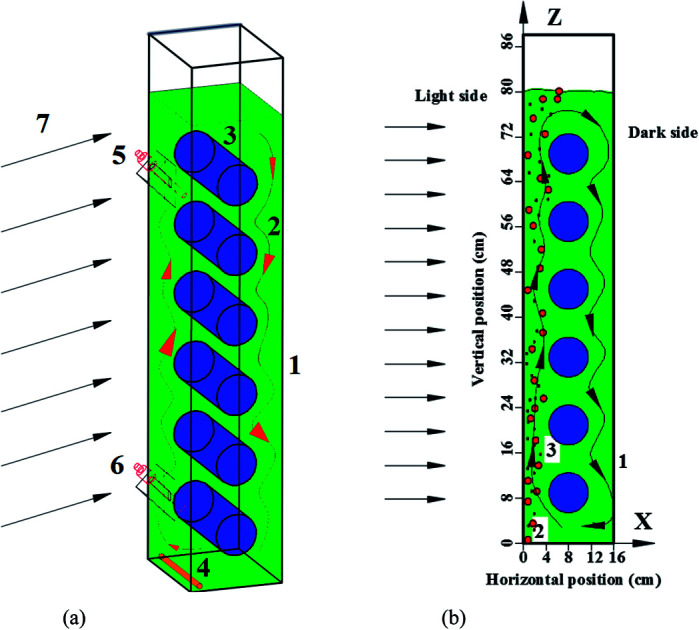

## 1. Introduction

CO_2_ is a greenhouse gas that mainly causes global warming and contributes to the formation of hostile environments. CO_2_ biological elimination by photosynthetic microalgae is a crucial way to mitigate CO_2_ from different sources, including the atmosphere and industrial exhaust gases, especially flue gas of coal-fired power plants.^1^ Lipid production from microalgae was also been optimized for new energy developments.^2,3^ Microalgae cultivation is vital in the utilization of microalgal biomass. Various types of bioreactor have been extensively utilized for algal culture, such as a raceway pond, flat panel bioreactors, and tubular reactors. Particularly, flat panel bioreactors have many advantages, such as a large illuminated surface, suitability for outdoor cultivation, good productivity, and being easy to clean.^4^ An appropriate mixed multiphase flow state in bioreactors is pivotal to supply CO_2_ efficiently, eliminate produced oxygen, provide alternate periods of light/dark (L/D), equably distribute nutrients, and avoid cell sedimentation.^5^ The influence of L/D cycles with different light intensities on the growth of microalgae has been researched.^6–9^ The vivid characterization of flow field in photobioreactors *via* experiment is difficult and costly to achieve.^10^ Developments of computational fluid dynamics (CFD) and the availability of more powerful computers have paved the way for the modeling and designing of a bioreactor.^11,12^ Soman *et al.* (2015) designed a bioreactor that combined flat plate bioreactors and airlift, and then further studied the superior liquid circulation properties of the bioreactor using CFD.^13^ It was proved that the design had a better surface to volume ratio and hydrodynamic properties. Kommareddy *et al.* (2017) further simulated the algal growth and hydrodynamic properties in the same bioreactors.^14^ Massart *et al.* (2014) established and validated a CFD hydrodynamic model for a flat-panel airlift bioreactor.^15^ In this respect, experimental water flow rates and the liquid circulation in the riser of the reactor were compared with the CFD solution results. However, these cases were simple combinations of airlift and flat plate bioreactors. So the problems of airlift bioreactors still existed in these design, such as low turbulent kinetic energy and low horizontal velocity in the downcomer.^16^ Moreover, microalgal cells movement and L/D cycle in the bioreactor were not investigated. New structure that can overcome the drawbacks of usual airlift flat panel photobioreactors should be designed and investigated.

The CO_2_ mass transfer performance of an airlift flat-plate bioreactor with flat baffle and waved baffle was studied by Chen *et al.* (2016)^17^ through the numerical and experimental methods. The results showed that the downcomer-to-riser cross-sectional area ratio played a major role on the mass transfer behavior of flat-plate airlift bioreactor. However, this research only studied the gas mass transfer in the reactor. The algal movement and L/D cycle of microalgal cells were not investigated. Moreover, algal cultivation validation was not performed. The flat-plate PBRs equipped with internal mixers was developed and further optimized its structure parameters using CFD by Huang *et al.* (2015).^18^ The maximum cell concentration and biomass productivity were 11.3% and 22.2% higher than those in the archetype. However, microalgal cells movement and L/D cycle in the bioreactor were not investigated. Novel baffles named HTTP baffles that produce vortices to improve the fluid velocity between light and dark areas in a flat-panel bioreactor were developed by Yang *et al.* (2016).^19^ Fluid velocity between light and dark areas increased from ∼0.9 cm s^−1^ to ∼3.5 cm s^−1^. Biomass yield increased by 70% with the enhanced flashing light effect. However, microalgal cells movement and L/D cycle in the new design remained unexplored due to the limitations of experimental measurement method.

In the present study, the movement of algal cells in the vortex flow field produced by horizontal baffles was analyzed through CFD. The cell L/D cycle period, fraction of time that the microalgal cell was exposed to light zone (light time fraction), and the horizontal fluid velocity were investigated at different gas aeration rates and microalgal concentrations. The results demonstrated that horizontal baffles can shorten the L/D cycle period of the microalgal cells and thereby improving the microalgal growth rate in flat panel bioreactor.

## 2. Materials and methods

### 2.1 Geometries of the flat-panel reactor and horizontal baffles

**Fig. 1:**
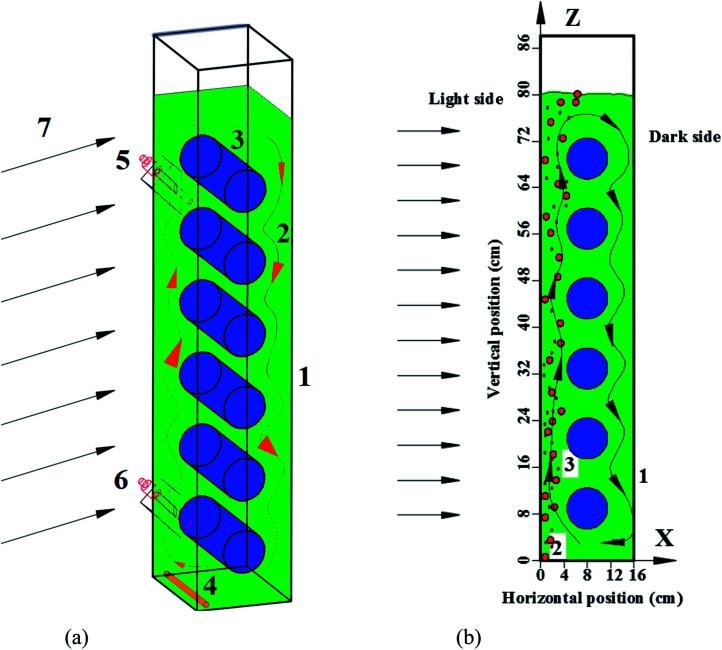
Schematic of a panel bioreactor with horizontal baffles. (a) Schematic of photobioreactor. (1) Panel bioreactor, (2) microalgae fluid with flow direction arrows, (3) horizontal baffles, (4) porous gas aerator, (5 and 6) two online precise pH probes or dissolved oxygen probes, (7) continuous artificial light, (b) side view of photobioreactor.

Panel bioreactor (PBR) schematic with horizontal baffles was showed in Fig. 1. It is 20 cm long, 16 cm wide, and 90 cm high. The diameters of the horizontal baffles are 7 cm with the length of 20 cm. The axis of the lowest horizontal tube is placed 8 cm from the left wall and 9 cm above the bottom of the PBR. Five more horizontal baffles have the same *x*-axis coordinates and the same axis distance of 12 cm on the *y*-axis direction.

### 2.2 Flow simulation in the flat-panel reactor

The PBR was 3D meshed using ANSYS ICEM CFD 15.0 (64 bit), and the simulation was conducted with ANSYS FLUENT 15.0 (64 bit). The Eulerian two-phase model was applied because using the multiphase model is unavoidable while bubbles occur in the photobioreactors. A standard *k-ε* model was chosen with first-order exactness to describe the turbulent flow behavior inside the PBRs. In this model, turbulent dispersion force and gas–liquid interphase drag force were considered. The outer walls and the internal structures of the PBRs were set as no-slip boundary conditions to water. The outlet was set as degassing boundary, representing that only the gas in the dispersed phase could escape from the surface and the continuous phase could not go through the top surface.^18^ The time step for the transient flow field computation was set as 0.004 s. To confirm grid independency, three scale grids (532 525; 904 944; and 1 174 084) were used. Small difference was found between the computed values of and 1 174 084 cells. So, the mesh with 904 944 cells was adopted for all the cases.

### 2.3 Fluid velocity and L/D cycle period calculation

Fluid velocity and L/D cycle period were calculated according to the result of simulation. The vertical fluid velocity (*V*_*z*_) was calculated with the velocity of two lines on the *z*-axis direction (*V*_*z*_1__ and *V*_*z*_2__). Line 1 was from point (*X* = −70 mm, *Y* = 0 mm, *Z* = 100 mm) to point (*X* = −70 mm, *Y* = 0 mm, *Z* = 700 mm), and line 2 was from point (*X* = 70 mm, *Y* = 0 mm, *Z* = 100 mm) to point (*X* = 70 mm, *Y* = 0 mm, *Z* = 700 mm). Thus, *V*_*z*_ = (*V*_*Z*_1__ + *V*_*Z*_2__)/2. The horizontal fluid velocity (*V*_*x*_) was calculated with the velocity of five lines on the *x*-axis direction. Line *i* (*i* = 1, 2, 3, 4, 5) was from point (*X* = −80 mm, *Y* = 0 mm, *Z* = 15 mm + 120 × *i* mm) to point (*X* = −80 mm, *Y* = 0 mm, *Z* = 15 mm + 120 × *i* mm). Thus, 
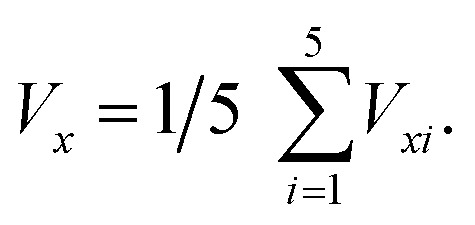
 The bottom of the PBR was set as (0, 0, 0), as described in Fig. 1a. A total of 1000 simulated particles were injected from two entrance ports; the two coordinates of the ports were (60 mm, 0 mm, 10 mm) and (−60 mm, 0 mm, 700 mm). The particle diameter used for the algal cells was 5 μm with a density of 1000 kg m^−3^. The maximum particle tracking time was set to 60 s. Discrete random walk model,^20^ drag force 
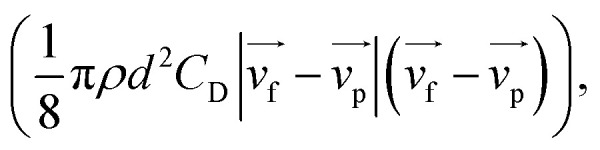
 and pressure gradient force 
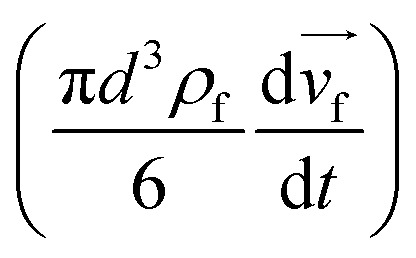
^21^ were considered during the simulation, where *d* is the particle diameter, *ρ* is its density, and *ν⃗* is the velocity vector, with the subscripts p stands for particle and f for the fluid (the continuous phase).

The spatial position of each spherical particle was recorded every 0.1 s. The L/D cycle period (*T*_av_^*i*^) of a microalgae cell was defined and calculated following the method described by Huang *et al.*^22^ The average L/D cycle period^10^ of each cell was used to calculate the average L/D cycle period of the entire population (*T*^p^_av_). 
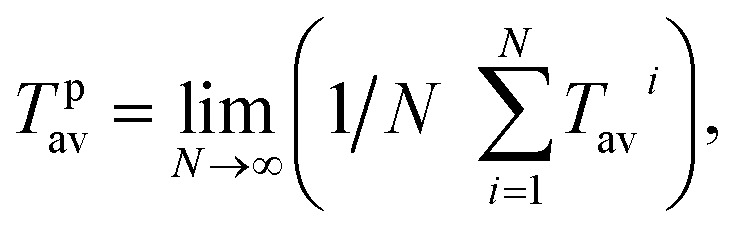
 where *N* is the number of the cells. Flashing light frequency (*f*) and light time fraction were defined as *f* = 1/*T*^*i*^_av_ and 
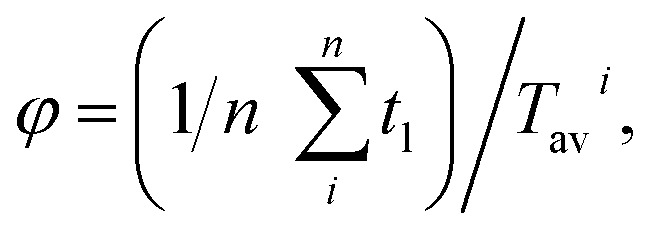
 respectively. All the parameters above were processed by MATLAB R2012b (64 bit) and Microsoft Office Excel.

### 2.4 Determination of critical depth between light and dark zone under various microalgal concentrations

A glass cylinder (diameter = 10 cm) was filled to different depths with the microalgal solution. A photosensitive electrode (GLZ-C, Zhejiang Top Instrument Co., Ltd. China) was installed on the bottom of the cylinder to record light intensity, and illumination intensity (*I*) detected by photosensitive electrode was recorded as fluid depths increasing in 1 cm increments. *I*_0_, which was the illumination intensity without microalgal solution, was recorded as 530 μmol m^−2^ s^−1^. In this study, the critical *I* of *Chlorella* was set as 96.84 μmol m^−2^ s^−1^ according to the report,^23^ and the depth corresponding to this critical *I* was defined as critical depth. In the PBR, the region where *I* was lower than the critical *I* or depth was bigger than the critical depth was defined as dark zone, and the remaining region was defined as light zone. Five concentrations of microalgal solution (*C*_*i*_ = 0.28, 0.56, 0.85, 1.1, 1.7 g L^−1^) were tested. Microalgal biomass were centrifugation at 8000 rpm for 5 min and then dried under 90 °C for 24 h to test microalgal concentrations by gram per liter.

### 2.5 Experimental measurement

Velocity magnitude was tested by using the miniature ultrasonic doppler velocimeter (Boyida Technology Co., Ltd., China) to validate the correctness of the simulation results for velocity. Solution-phase mixing time were calculated according to the method of Mendoza *et al.* (2013).^24^ Water was employed as test fluid during the measurement. Initially, the water pH was adjusted to 4.0 ± 1 by adding chlorhydric acid (35%, w/v). Then, 0.20 ml NaOH solution (12 mol L^−1^) per liter of water was added as alkalinity tracer. The time was recorded when the alkalinity tracer was added. The mixing time is the required time for pH variations reaching to lower than 5% of the final stable value. The pH probes were used to measure the response to the pH pulse at two positions in this PBR. The overall volumetric mass-transfer coefficient *K*_L_*a*_L_ was measured by the method of Sierra *et al.*(2008).^25^ Water in the PBR was alternately aerated with air and N_2_. N_2_ and air aeration rates were controlled by mass flow meter (SevenstarCS200, China). Then, mass-transfer coefficient was calculated according to the formula: d*C*_L_/d*t* = *k*_L_*a*_L_(C* − *C*_L_), where C* was the saturation concentration of dissolved oxygen. Dissolved oxygen probes (Mettler Toledo, InPro6850i/12/120) and pH probes (Mettler Toledo, InPro3253i/SG/120) were connected to transmitters and data acquisition software (i-7017fc, ICP DAS, Taiwan). Every 0.1 s, measurements were automatically recorded.

### 2.6 Microalgal cultivation

Microalgae mutant *Chlorella* PY-ZU1 was cultivated with Bristol's solution (also called soil extract, SE) and measured by the same method described by Cheng *et al.* (2013).^26^*Chlorella* PY-ZU1 was cultivated in the flat-plate PBR under 23 °C with continuous illumination of 530 μmol m^−2^ s^−1^. 15% CO_2_ was continuously aerated into the culture medium with 0.02 vvm flow rate.

## 3. Results and discussion

### 3.1 L/D cycle periods in the PBR with horizontal baffles

**Fig. 2:**
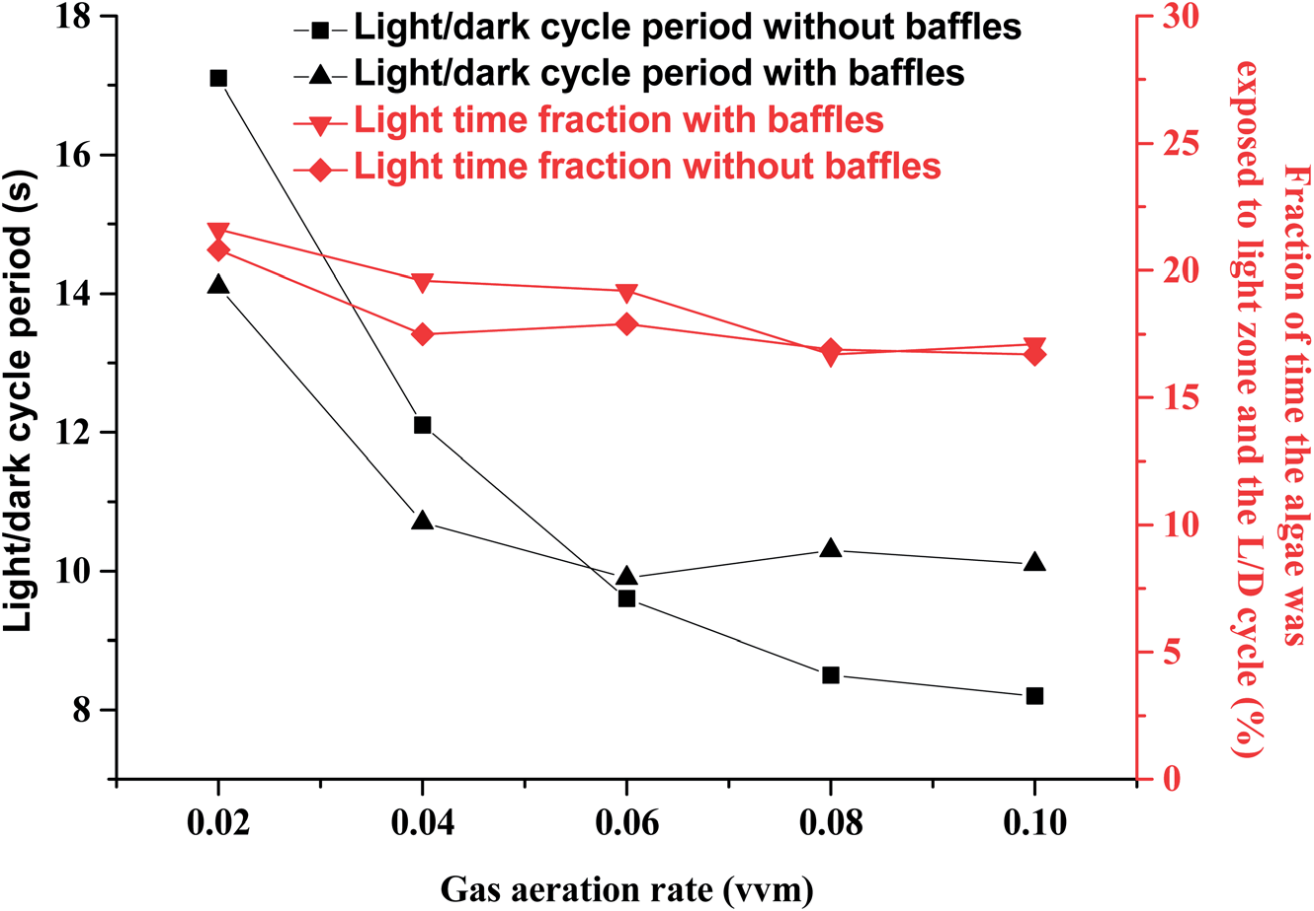
Effects of gas aeration rate on light/dark cycle period in a panel bioreactor with horizontal baffles.

**Fig. 3:**
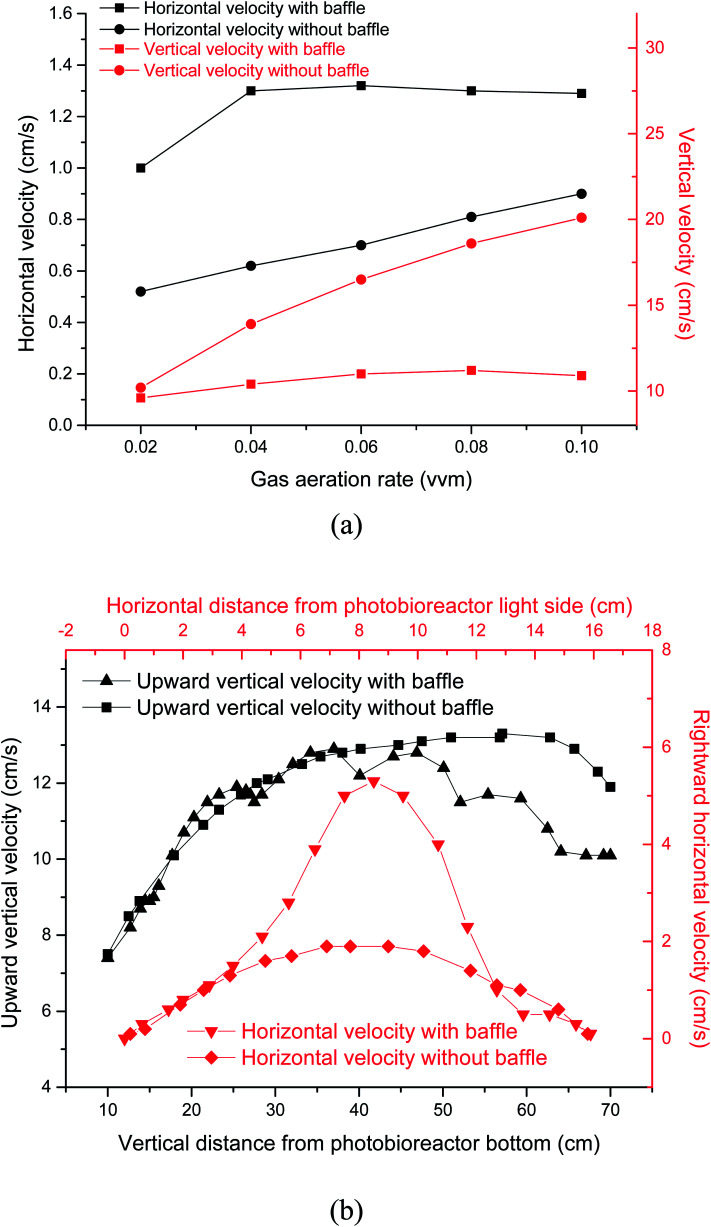
Vertical velocity and horizontal velocity of fluid in a panel bioreactor with horizontal baffles. (a) Vertical velocity and horizontal velocity under different gas aeration rate (b) vertical velocity and horizontal velocity at different position in a panel bioreactor under gas aeration rate of 0.02 vvm.

Light transmission in microalgal solution is limited. Microalgal cells grow slowly in the deep position where light can not reach. Flashing light effect in microalgae cultivation field was regarded as periodic exposure of microalgae to light or rapid travel between dark and light zones. Flashing-light effect can be characterized by L/D cycle period. When microalgal concentration was 0.85 g L^−1^, the critical depth from light zone to dark zone was test to be 2 cm. Fig. 2 showed the effects of gas aeration rate on light/dark cycle period in a PBR with or without horizontal baffles under a microalgal concentration of 0.85 g L^−1^, which was analyzed according to the simulation result. While the air flow rate increased from 0.02 vvm to 0.04 vvm, the L/D cycle period decreased from 14.1 s to 10.7 s with the horizontal baffles, and decreased from 17.1 s to 12.1 s without the horizontal baffles. The addition of horizontal baffles decreased the L/D cycle period by 17.4% under 0.02 vvm air aeration rate. The flashing-light effect of the microalgae from dark area to light area was improved. Microalgal growth rate can be obviously improved with the enhanced flashing light effect.^27^Fig. 3b showed the vertical velocity and horizontal velocity of fluid in a PBR with or without horizontal baffles, which was obtained from the simulation result. As the aeration rate increased from 0.02 vvm to 0.04 vvm, horizontal fluid velocity increased from 1.0 cm s^−1^ to 1.3 cm s^−1^ with the horizontal baffles, and increased from 0.52 cm s^−1^ to 0.62 cm s^−1^ without the horizontal baffles [Fig. 3a]. The velocity magnitudes in the PBRs with and without horizontal baffles were measured using a miniature ultrasonic Doppler velocimeter. When the aeration rate was 0.02 vvm, the test average velocity magnitudes were 0.95 cm s^−1^ with horizontal baffles and 0.48 cm s^−1^ without horizontal baffles, and the differences from the simulation results were 5% and 7.7%, respectively. This result demonstrated that the simulation result was acceptable.

With the horizontal baffles, small scale vortex flow was developed in the PBR, around one horizontal tube baffle, or between two baffles. In the presence of horizontal baffles under air aeration rate of 0.02 vvm, the horizontal fluid velocity significantly increased by 1.8 times especially in the middle of the PBR. Thus, culture fluid can be quickly moved from dark area to light area. Only a large vortex flow was generated within PBR without horizontal baffles. Then, fluid flow direction mainly changed at the bottom and top of the PBR.^28^ The major part of the microalgal culture fluid cannot move quickly to the other side in the central section of the PBR. Several studies reported that flashing light effect of the microalgae from dark area to light area can enhance photosynthesis and improve the quality and quantity of microalgal biomass. For that reason, it is significant to consider the integration of flashing light effect into microalgal cultivation systems.^17^

The vertical fluid velocity increased from 9.6 cm s^−1^ to 10.4 cm s^−1^ and from 10.2 cm s^−1^ to 13.9 cm s^−1^ as the gas aeration rate increased from 0.02 vvm to 0.04 vvm with and without the horizontal baffles. Vertical fluid velocity decreased by 25% when the horizontal baffles were used under 0.04 vvm air aeration. Longer bubble residence time can be achieved with a slower vertical fluid velocity. The CO_2_ utilization efficiency was improved as more CO_2_ was dissolved into the culture fluid.

Fluid movement was obviously affected by horizontal baffles, especially in the upper section of the PBR [Fig. 3b] as parts of air bubbles were blocked by the horizontal baffles. The vertical fluid velocity was not increased further as aeration rate increased from 0.06 vvm to 0.1 vvm with the horizontal baffles. So the L/D cycle period was not decreased further. Vertical and horizontal fluid velocities increased from 16.5 cm s^−1^ to 20.1 cm s^−1^ and from 0.7 cm s^−1^ to 0.9 cm s^−1^, respectively, while gas aeration rate increased from 0.06 vvm to 0.1 vvm without horizontal baffles. A high intensity turbulent region was developed within the reactor. Hence, the L/D cycle period decreased from 9.6 s to 8.2 s while gas flow rate increased from 0.06 vvm to 0.1 vvm.

Light zone was only 2 cm depth from the light direction with a microalgal concentration of 0.85 g L^−1^. So light time of algal cell was decreased with the increased vertical fluid velocity while gas aeration rate was increased from 0.02 vvm to 0.1 vvm. Thus, the light time fraction slightly decreased from ∼21% to ∼17% (Fig. 2). Flue-gas (especially from a coal-fired power plant) aeration rate is restricted because it contains NO_*x*_ and SO_*x*_,^29^ high flashing light frequency can be obtained with horizontal baffles at a low gas aeration rate.

### 3.2 L/D cycle periods under different microalgal concentration

**Fig. 4:**
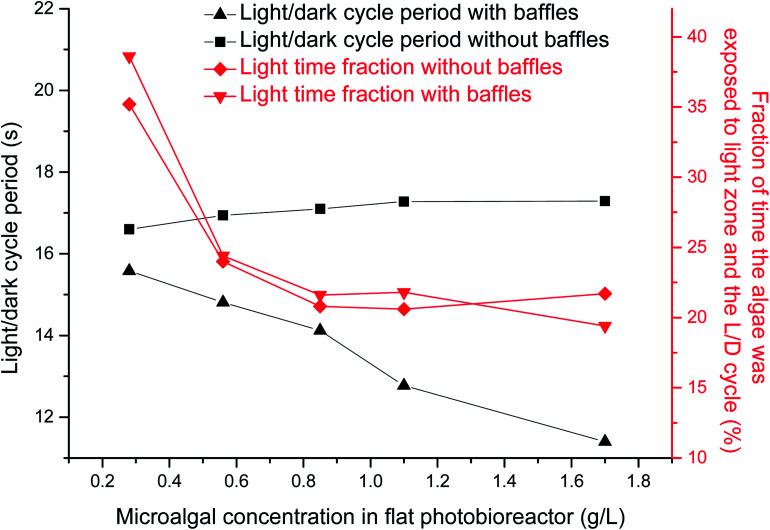
Effects of microalgal concentration on light/dark cycle period in a panel bioreactor with horizontal baffles.

The L/D cycle period time was calculated based on different microalgal concentrations under 0.02 vvm gas aeration rate. The critical depth was decreased from 5 cm to 1 cm when the microalgal concentration was increased from 0.28 g L^−1^ to 1.7 g L^−1^. L/D cycle period decreased from 15.6 s to 11.4 s and the light time fraction decreased from 39% to 17% when the microalgal concentration was increased from 0.28 g L^−1^ to 1.7 g L^−1^ in the presence of horizontal baffles (Fig. 4). The cross-sectional flow area was periodically changed when horizontal baffles were used. Air was aerated close to right side of the wall (Fig. 1b). Small scale vortex flow was developed in the plate reactor, around one horizontal tube baffle, or between two of the horizontal baffles. Fluid fluctuations increased when the critical depth line moved to the right side of the wall. Thus, the L/D cycle period decreased by 27% as the critical depth was decreased from 5 cm to 1 cm.

The L/D cycle period was ∼17 s and light time fraction decreased from 35% to 22% when the microalgal concentration was increased from 0.28 g L^−1^ to 1.7 g L^−1^ in the absence of horizontal baffles (Fig. 4). Flow direction of fluid mostly changed at the bottom and top of the PBR. Most of the microalgae fluid can not move quickly from dark area to light area in center section of the PBR. Movement of particles inside the PBR was relatively uniform; thus, the L/D cycle period time was not shortened with the increase of microalgal concentration.

**Fig. 5:**
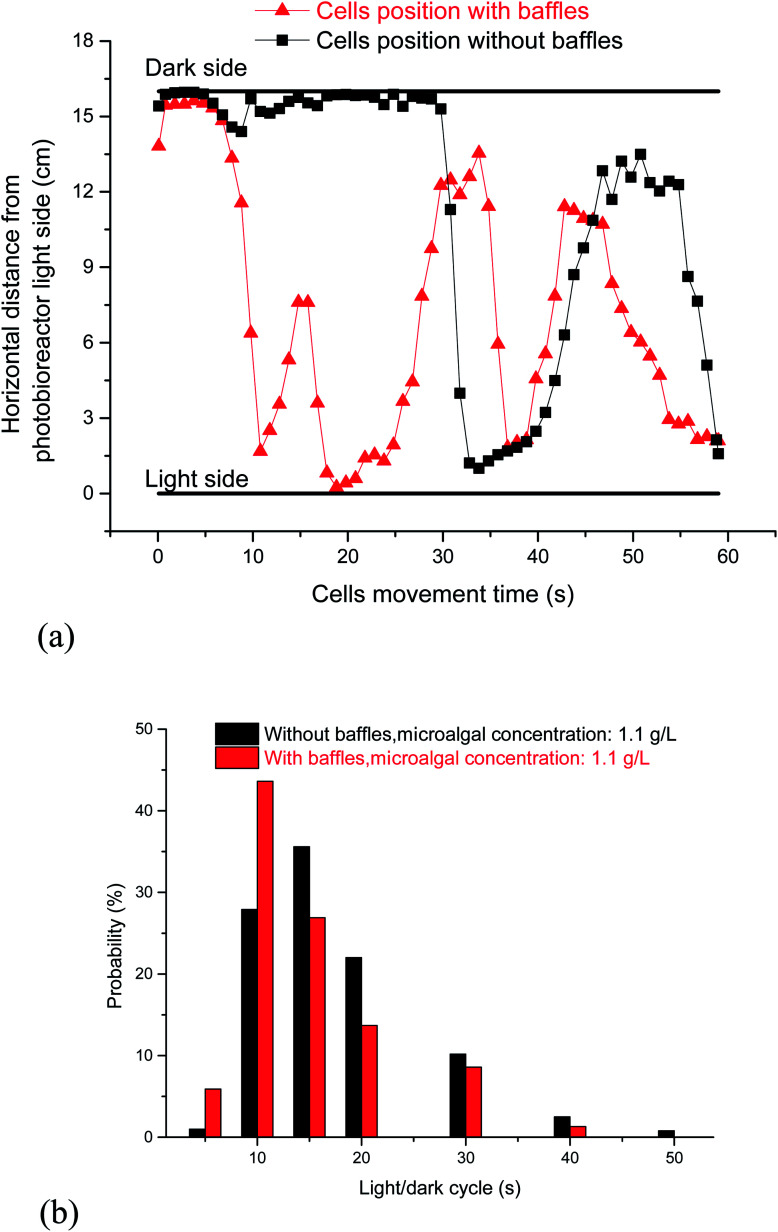
Effects of horizontal baffles on cell flow trajectory in a panel bioreactor. (a) Cells flow trajectory between light side and dark side; (b) probability of light/dark cycle period.

The L/D cycle period, which was based on different microalgal concentrations, was the same at different fluid depths. The dark area comprises roughly 90% of the cycle when the microalgal concentration was 1.1 g L^−1^. Microalgal cells cannot timely move from one side to the other side in the PBR without horizontal baffles (Fig. 5a). Small light dark cycle period was good for cells growth. Most L/D cycle periods were longer than 5 s, and the probability of the L/D cycle period with 5–10 s was 27.9% (Fig. 5b). The fluid flow direction was changed quickly, vortex flow fields were produced, and the vertical fluid velocity increased from 0.52 cm s^−1^ to 1.0 cm s^−1^ when the horizontal baffles were used. Consequently, the cell was quickly moved from dark area to light area in the PBR (Fig. 5a). The probability of the L/D cycle period with 0–5 s increased from 1% to 5.9%, and the probability of the L/D cycle period with 5–10 s increased from 27.9% to 43.6% (Fig. 5b), indicating an increase of 56% when the horizontal baffles were used. The result showed that the cells has a bigger probability to gain a small light dark cycle period in PBR with horizontal baffles than that in PBR with baffles.

### 3.3 Solution mixing and mass transfer in the PBR with horizontal baffles

**Fig. 6:**
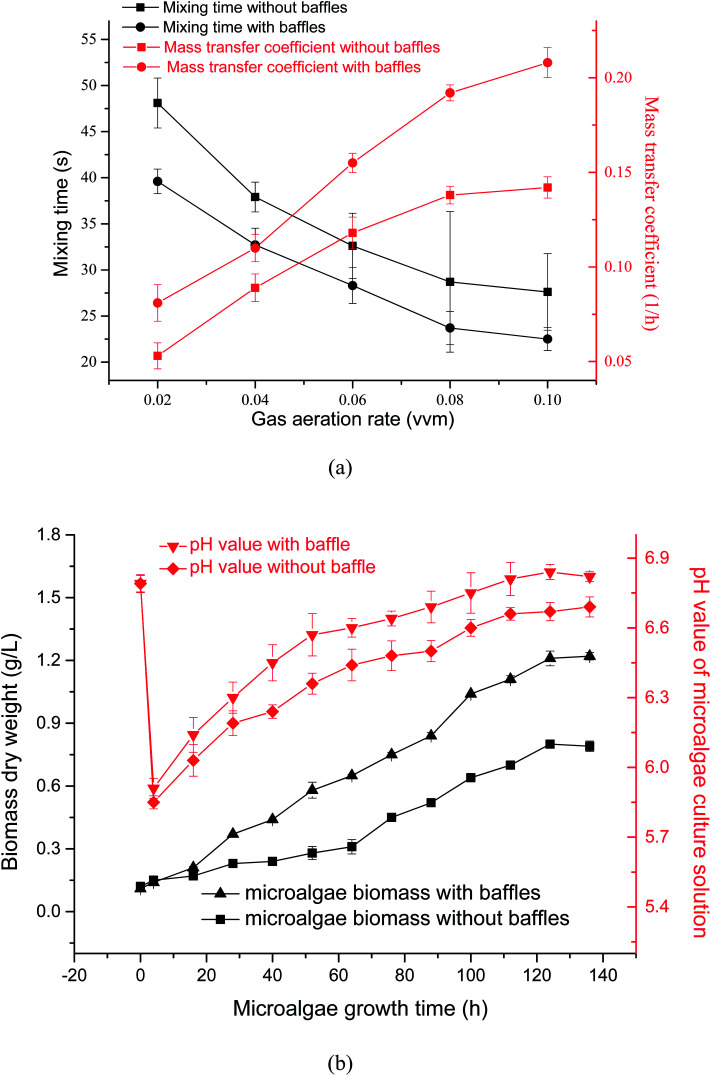
Microalgal growth in an optimized flow field of a panel bioreactor with horizontal baffles. (a) Effects of gas aeration rate on mixing time and mass transfer; (b) effects of horizontal baffles on pH and microalgal growth.

Effects of gas aeration rate on mass transfer coefficient and mixing time in PBR with or without the horizontal baffles were tested by experiment [Fig. 6a]. When the gas aeration rate was increased from 0.02 vvm to 0.1 vvm, mass-transfer coefficient decreased from 48.1 s to 27.6 s without the horizontal baffles, and decreased from 39.6 s to 22.5 s with the horizontal baffles. When the horizontal baffles existed, mixing time decreased by 13% under 0.06 vvm gas aeration rate. The PBR mixing efficiency improved (bigger horizontal velocity) while horizontal baffles were used. Appropriate mixing condition allowed even nutrient distribution in the culture medium and accelerated microalgal growth.^30^

When air aeration rate was increased from 0.02 vvm to 0.1 vvm, mass-transfer coefficient increased from 0.053 h^−1^ to 0.142 h^−1^ without the horizontal baffles, and increased from 0.081 h^−1^ to 0.208 h^−1^ with the horizontal baffles. The residence time of rising bubbles was higher in the culture solution because that solution vertical velocity decreased by 18% under 0.06 vvm gas aeration rate. Therefore, mass transfer coefficient increased by 31% under 0.06 vvm gas aeration rate, thus leading to higher mass transfer and accelerated CO_2_ dissolution.^31,32^

### 3.4 Increased microalgal biomass yield in the presence of horizontal baffles

Effects of horizontal baffles on microalgal growth rate and pH values *versus* time with 15% CO_2_ under a aeration rate of 0.02 vvm were illustrated in Fig. 6b. The pH of SE culture decreased quickly from 6.8 to 5.8 during the first 4 h because CO_2_ quickly dissolved into the medium. The culture pH increased slowly after 4 h, this increasing tendency of pH owing to the CO_2_ uptake by microalgae. The fluid flow direction was changed quickly, vortex flow fields were produced, and the vertical fluid velocity increased from 0.52 cm s^−1^ to 1.0 cm s^−1^ when the horizontal baffles were used.

Consequently, the cell was quickly moved from dark area to light area in the PBR (Fig. 5a). The probability of the L/D cycle period with 5–10 s increased by 56% from 27.9% to 43.6% when the horizontal baffles were used. So the L/D cycle period decreased by 17.4% under an air aeration rate of 0.02 vvm when horizontal baffles were added. It is proposed that mixing has two separate but synergistic effects, for example, it not only moves the microalgal cells through a L/D cycle, but also decreases the boundary layer, which increases the rate of exchange through the cell wall of nutrients and metabolites. Thus, more nutrients can be uptake and light can be utilized more efficiently, so the biomass yield is increased.^33^ The biomass yield in the fifth day increased by roughly 51% in comparison with the condition without baffles at a same aeration rate.

## 4. Conclusion

Hydrodynamic and flashing effect of a panel bioreactor with horizontal baffles were investigated by CFD simulation. The L/D cycle period decreased by 17.5% and the probability of the L/D cycle period within 5–10 s increased by 56% when horizontal baffles were used under 0.02 vvm gas aeration rate. Experiments confirmed the enhanced flow field in the bioreactor. Mixing time decreased by 13% and mass-transfer coefficient increased by 31% under 0.06 vvm gas aeration rate. The microalgal biomass yield increased by 51% with the same light intensity. The optimized width of the PBR under different light intensities can be further investigated to increase the microalgal biomass yield per unit area.

## Conflicts of interest

There are no conflicts to declare.

## Supplementary Material

RA-008-C8RA02863J-s001
