# Supplementary material for: Improving microalgal growth by strengthening the flashing light effect simulated with computational fluid dynamics in a panel bioreactor with horizontal baffles
Source: RSC Adv. 2018 May 23;8(34):18828–36. doi: 10.1039/c8ra02863j (PMC9080617; doi:10.1039/c8ra02863j)
Supplement: RA-008-C8RA02863J-s001 [file RA-008-C8RA02863J-s001.pdf]

Electronic Supplementary Material (ESI) for RSC Advances.
This journal is © The Royal Society of Chemistry 2018

Support Materials

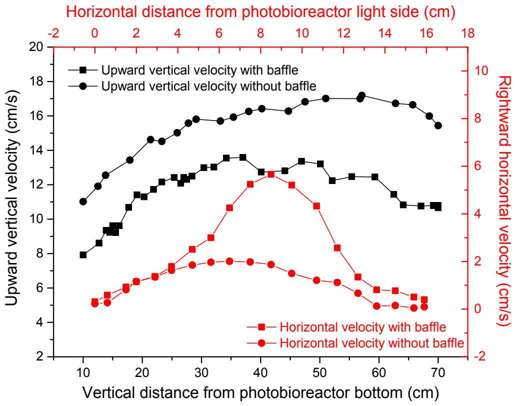

□a□

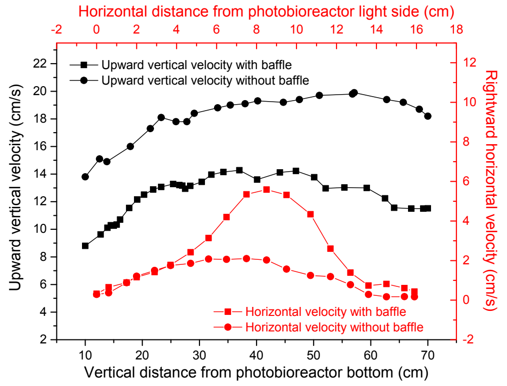

(b)

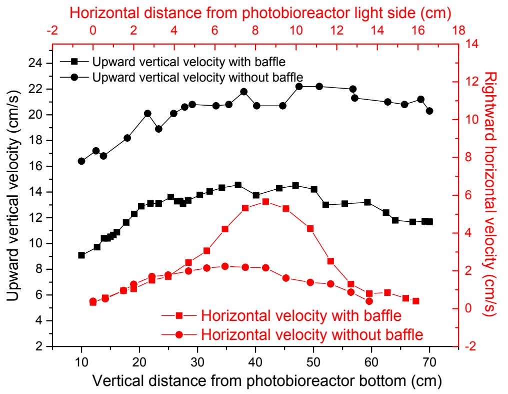

(c)

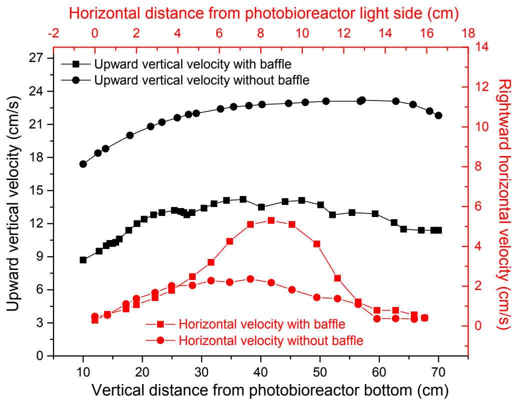

(d)

Fig.1 Vertical velocity and horizontal velocity at different position in a panel bioreactor under various gas aeration rate. (a) 0.04 vvm, (b) 0.06 vvm, (c) 0.08 vvm, (d) 0.10 vvm.
